# Calibration of the EBT3 Gafchromic Film Using HNN Deep Learning

**DOI:** 10.1155/2021/8838401

**Published:** 2021-01-31

**Authors:** Liyun Chang, Shyh-An Yeh, Sheng-Yow Ho, Hueisch-Jy Ding, Pang-Yu Chen, Tsair-Fwu Lee

**Affiliations:** ^1^Department of Medical Imaging and Radiological Sciences, I-Shou University, Kaohsiung 82445, Taiwan; ^2^Department of Radiation Oncology, E-Da Hospital, Kaohsiung 82445, Taiwan; ^3^Graduate Institute of Medical Sciences, Chang Jung Christian University, Tainan 71101, Taiwan; ^4^Department of Radiation Oncology, Chi Mei Medical Center, Tainan 71004, Taiwan; ^5^Department of Radiation Oncology, Tainan Sin-Lau Hospital, Tainan 70142, Taiwan; ^6^Medical Physics and Informatics Laboratory of Electronics Engineering, National Kaohsiung University of Science and Technology, Kaohsiung 80778, Taiwan; ^7^PhD Program in Biomedical Engineering, Kaohsiung Medical University, Kaohsiung 80708, Taiwan

## Abstract

To achieve a dose distribution conformal to the target volume while sparing normal tissues, intensity modulation with steep dose gradient is used for treatment planning. To successfully deliver such treatment, high spatial and dosimetric accuracy are crucial and need to be verified. With high 2D dosimetry resolution and a self-development property, the Ashland Inc. product EBT3 Gafchromic film is a widely used quality assurance tool designed especially for this. However, the film should be recalibrated each quarter due to the “aging effect,” and calibration uncertainties always exist between individual films even in the same lot. Recently, artificial neural networks (ANN) are applied to many fields. If a physicist can collect the calibration data, it could be accumulated to be a substantial ANN data input used for film calibration. We therefore use the Keras functional Application Program Interface to build a hierarchical neural network (HNN), with the inputs of net optical densities, pixel values, and inverse transmittances to reveal the delivered dose and train the neural network with deep learning. For comparison, the film dose calculated using red-channel net optical density with power function fitting was performed and taken as a conventional method. The results show that the percentage error of the film dose using the HNN method is less than 4% for the aging effect verification test and less than 4.5% for the intralot variation test; in contrast, the conventional method could yield errors higher than 10% and 7%, respectively. This HNN method to calibrate the EBT film could be further improved by adding training data or adjusting the HNN structure. The model could help physicists spend less calibration time and reduce film usage.

## 1. Introduction

In addition to dose painting, various strategies of radiation therapy with steep dose gradients are used to deliver a nonuniform dose to a clinical target with reduced toxicity to normal tissues [1, 2]. To ensure both spatial and dosimetric accuracy, quality assurance (QA) is vital for treatment centers. Several two-dimensional dosimetry tools have been introduced to expedite this QA, including portal dosimetry devices [3, 4], matrix detectors [5–9], and film dosimeters [4, 10–12]. Of these, the Gafchromic EBT film is widely used, largely due to its self-development characteristic, near dose-to-water equivalence [13], high spatial resolution, rereadability, relatively uniform dose-response across a wide range of photon energies [2, 11, 14], and inexpensive techniques for read-out using commercially available flatbed document scanners [15].

Several generations of the Gafchromic film have been developed, but only EBT2 and EBT3 film models are recommended by Ashland for verifying all beam-modulated techniques [16]. This is because spatial nonhomogeneity is corrected by its yellow marker dye [15, 17–22], so it is less sensitive to the visible spectrum, and it is available for repeated scans [23, 24]. With the matte polyester substrate to avoid the formation of Newton's rings [25–28], the EBT3 film has active layer composition and dosimetric properties similar to EBT2 [26], with insignificant side dependence of the film [29]. Based on the Ashland report, the effective atomic numbers of EBT2 and EBT3 films are around 6.8 and 7.3, which is approximately water equivalent, increasing their suitability for patient dosimetry [24, 30].

However, the calibration responses and the fitting parameters change due to sensitivity variations between film lots and the so-called film “aging effect” that changes film sensitivity with shelf life [31–35]. The film aging effect can be diminished if the background is subtracted using the net optical density, a conventional calibration method; but periodical recalibration (e.g., once per quarter) is recommended [33, 35].

Film calibration can be done by extracting the net optical density [36], the pixel value [24], and the inverse transmittance [37] appropriate for the delivered dose with the adequate equations. Recently, Zhuang et al. used the pytorch artificial neural network (ANN) platform (https://pytorch.org/) with inputs of optical densities for calibrating different EBT3 lots [38]. Zhuang subsequently did a trial with 400 training inputs from 6 films, where each film had different lot numbers, and the mean square errors (MSE) of the test batches reached 18 cGy. In our study, a hierarchical neural network (HNN) was built using the Keras functional Application Program Interface (https://keras.io/guides/functional_api/). Hierarchical networks, based on a hierarchical organization, consist of several ANN subnets, each of which deals with a specific aspect of the input data. The subnet models with some input variables determine the overall training pattern [39]. HNN was previously used for survival analysis [40]. Here, it is used to find a solution for the film age effect and intralot variation.

## 2. Materials and Methods

Gafchromic EBT3 films from different lots were scanned by an Epson 10000XL scanner in a fixed portrait orientation to create 127 dpi tiff images before and after calibration delivery, referred to as prescan and postscan, respectively. Just before calibration delivery, a 6 MV photon beam from an Elekta Synergy accelerator was quickly calibrated at the depth of 5 cm (SSD 95 cm, field size 10^2^ cm^2^ and 1 cGy/MU) according to AAPM TG reports [41–44]. Then, the film was tightly sandwiched in a 30 cm cubic RW3 polystyrene phantom, and the cubic phantom was located above another 10 cm thick backup plates. The film plane was parallel to the beam central axis with its midline, the line longitudinally separated the film into two equal parts, oriented to be coincident with the central beam.

A dose in the daily treatment range was delivered to the film, and the film dose at midline was exactly calculated by the delivered MU and the verified percentage depth doses [24, 36, 37, 45–47]. After 24 hours, each film was rescanned with the same 127 dpi, and all the tiff format images were analyzed using the Matlab and Keras software. Lot No. 03211802 EBT3 films (Lot C) were exposed 17 times at different dates within 20 months for film calibration. The time interval between the 16^th^ and 17^th^ calibration was 4 months. The previous 11 and 16 times calibrations of lot C films were collected to be portion I and II training data with 2394 and 3762 inputs, respectively. The training data of portion II was used to manage the film aging effect since it needs longer collection, and the 17^th^ calibration film was used for the test data. Portion I was used for the intralot variation verification test by using lot A (lot No. 07191602) with 7 calibrations and lot B (lot No. 03071603) with 3 calibrations. For comparison with the developed HNN method, the conventional method is introduced below.

### 2.1. Conventional Method

The red-channel net optical density (R-NOD) of the calibration film can be written as

(1) $$\begin{array}{c} \text{RNOD}=\log_{10}\left[\frac{{\text{RPV}}_{\text{pre}}}{\text{R}{\text{PV}}_{\text{post}}}\right], \end{array}$$

where RPV_pre_ and RPV_post_ are the extracted red-channel pixel value (PV) from the prescan (background) image and the postscan image, respectively.

The R-NOD extracted from the midline of the film is fitted to the delivered dose using the power function

(2) $$\begin{array}{c} {\text{D}}_{\text{fit}}=a\times \text{RNOD}+b\times {\text{RNOD}}^{c}, \end{array}$$

where *D*_fit_ is the fitted dose; and *a*, *b* and *c* are fitting parameters. The fitting process was repeated twice, the first time with *a* and *b* not bound, but *c* bound between 1 and 3. After obtaining the fitted *c* value, it was rounded to the nearest tenth. Then, the second fitting process was started with the same parameter values as the first. The percentage error between the calculated dose and delivered dose, *E*_tr_, using the conventional method can be written as

(3) $$\begin{array}{c} E_{\text{tr}}=\left(D_{c}-D_{d}\right)/D_{d}\times 100\%, \end{array}$$

where *D*_*c*_ is the calculated dose by equation (2) and *D*_*d*_ is the delivered dose.

The films of the first calibration of lots A and B and the 16^th^ calibration of lot C were used to calculate the fitting parameters individually through the power function of equation (2). All the other films of lots A and B and the 17^th^ calibration of lot C were used for the verification test.

### 2.2. Deep Learning HNN Method

A hierarchical neural network (HNN) was built using the Keras deep learning Application Program Interface (API), written in Python and running on top of the machine learning platform TensorFlow. The input parameters for the HNN training are R-NOD, red-channel irradiated PV (R-IPV) extracted from the postscan image with the red-channel background PV (R-BPV) extracted from the prescan image, green-channel irradiated PV (G-IPV) with green-channel background PV (G-BPV), blue-channel irradiated PV (B-IPV) with blue-channel background PV (B-BPV), red-channel inverse transmittance (R-IT) with green-channel inverse transmittance (G-IT), and with blue-channel inverse transmittance (B-IT). The inverse transmittance (IT), *T*_*W*_, can be written as

(4) $$\begin{array}{c} T_{W}=\left(2^{16}-1\right)/\text{P}{\text{V}}_{W} \end{array}$$

where *W* represents one of the R, G, and B channels.

**Figure 1:**
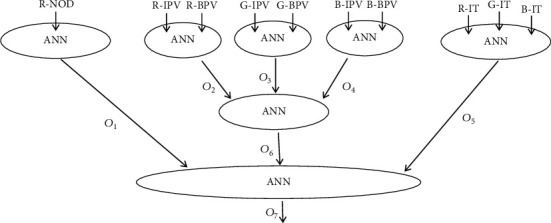
Simplified deep learning HNN frame using the Keras functional API for film-dose calibration.

Some of the input parameters may depend on each other; however, all have been used for film calibration with different techniques [24, 36, 37] since each has its own advantages. The red-channel PV has the highest sensitivity to the dose range of daily treatment, while the green-channel PV and blue-channel PV have higher dynamic responses to higher delivered doses [37, 48]. As the earliest used parameter, with many published papers, R-NOD was gradually replaced by the IT of the RGB used for the three-channel calibration technique [36, 37]. The three-channel background PV was intended to manage the film aging effect. These ten kinds of inputs were reorganized as five input groups: (1) R-NOD, (2) R-IPV/R-BPV, (3) G-IPV/G-BPV, (4) B-IPV/B-BPV, and (5) R-IT/G-IT/B-IT; as shown in Figure 1.

**Figure 2:**
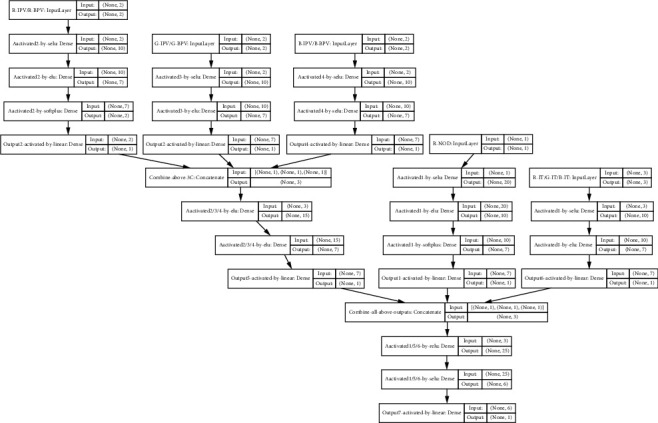
Detailed structure of the deep learning HNN.

This structure can be described with the functions *O*_1_, *O*_2_ ⋯ *O*_7_ : *O*_1_ = *f* (R-NOD), *O*_2_ = *f* (R-IPV, R-BPV), *O*_3_ = *f* (G-IPV, G-BPV), *O*_4_ = *f* (B-IPV, B-BPV), *O*_5_ = *f* (R-IT, G-IT,B-IT), *O*_6_ = *f* (*O*_2_, *O*_3_, *O*_4_), and *O*_7_ = *f* (*O*_1_, *O*_5_, *O*_6_), where *O*_1_(.) is approached with one input, 20 neurons in the 1^st^ hidden layer, 10 neurons in the 2^nd^ hidden layer, 7 neurons in the 3^rd^ hidden layer, and one output (i.e., model 1-20-10-7-1); *O*_2_(.) is approached with a model 2-10-7-2-1; *O*_3_(.) is approached with a model 2-10-7-1; *O*_4_(.) is approached with a model 2-10-7-1; *O*_5_(.) is approached with a model 3-15-7-1; *O*_6_(.) is approached with a model 3-10-7-1; and *O*_7_(.) is approached with a model 3-20-6-1.  Figure 2 illustrates the detailed structure.

“Selu,” “elu,” “relu,” “softplus,” and “linear” are activation functions. The initial weighting was set as a uniform, random number generator seed 435. The optimization algorithm “Adam” is used as an extension to stochastic gradient descent in place of classical stochastic gradient descent to update network weights more efficiently and steadily. Since the training deals with a multiple-regression problem, a mean squared error (MSE) objective function is optimized through the “Adam” optimizer. MSE is also a desirable metric that is used to evaluate performance of the model. The other two metrics used in this HNN are “mean absolute error” (MAE) and “accuracy.” Then, the fitting process was executing with batch size of 20 and 500 epochs. The validation split is 0.45; that is, 45% training data was held back for validation.

**Figure 3:**
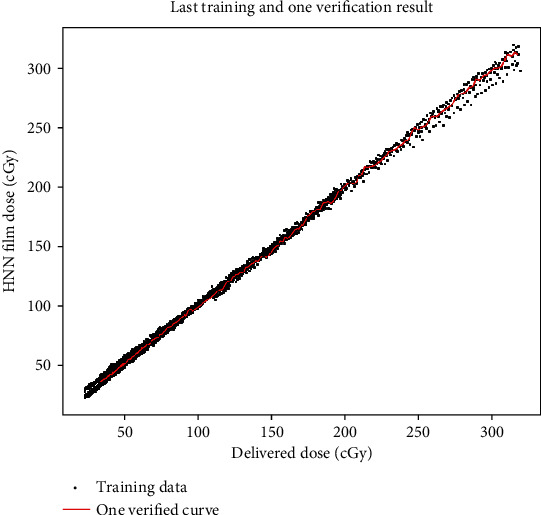
Training results and one training data curve for verification.

The number of hidden layers, neurons, and activation functions were systematically adjusted so all the calculated doses converged to be equal to the delivered doses, which can be examined through the value of MSE and MAE and the illustration of the delivered dose with the calculated dose. The training results using portion I films is shown in Figure 3, where the red line is one calibration data of portion I.

### 2.3. Intralot Verification Test

**Figure 4:**
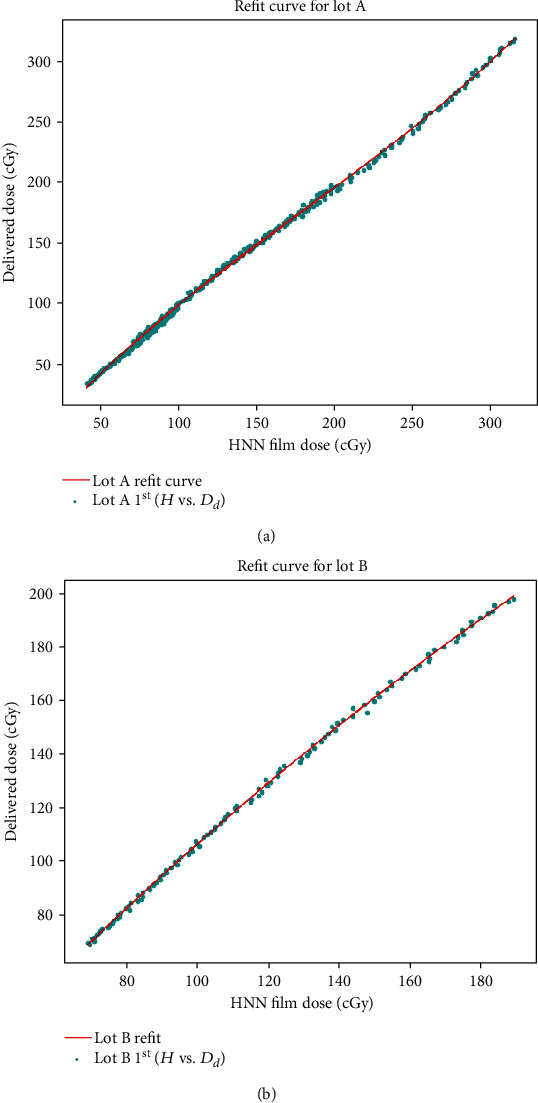
Refit of the HNN dose of the 1^st^ calibration films of (a) lot A and (b) lot B through equation (5).

Due to sensitivity variations between lots, the calculated dose through the trained HNN model for the 1^st^ calibration film of lot A and lot B film is found clearly away from the line, where calculated doses are equal to delivered doses. To make the lot C training results work for lots A and B, the calculated film doses of the 1^st^ calibration films of lots A and B through the trained HNN are refitted to the delivered dose *D*_*d*_ as below:

(5) $$\begin{array}{c} D_{\text{rfit}}=e\times H^{3}+f\times H^{2}+g\times \text{H}+k, \end{array}$$

where *D*_rfit_ is the fitted dose; *e*, *f*, *g*, and *k* are fitting parameters; and *H* is the calculated dose through the trained HNN. The fitting results are shown in Figure 4. equation (5) is then used to calculate the film doses of lots A and B.

### 2.4. Aging Effect Verification Test

The 17^th^ calibration film of lot C is used for the verification test of film aging. The dose of the 17^th^ verification film is calculated through the trained HNN that was executed by using the films of portion II, and the calculated dose is compared with the delivered dose. Here, the refit (equation (5)) is not performed, since the test lot and training lot are the same lot.

Applying the deep learning HNN method, the *H* value of the first calibration film of lots A and B was used to calculate the fitting parameters of equation (5), and all the other films of lots A and B and the 17^th^ calibration lot C film will be used for the verification test of the interlot and aging effect, respectively. The percentage error between the calculated dose and the delivered dose, *E*_hnn_, using the HNN method can be written as

(6) $$\begin{array}{c} E_{\text{hnn}}=\left(D_{\text{rd}}=D_{d}\right)/D_{d}\times 100\% \end{array}$$

where *D*_rd_ will be the film doses calculated through equation (5) for lot A and lot B and *D*_rd_ = *H* for the lot C film of the 17^th^ calibration.

## 3. Results and Discussion

**Figure 5:**
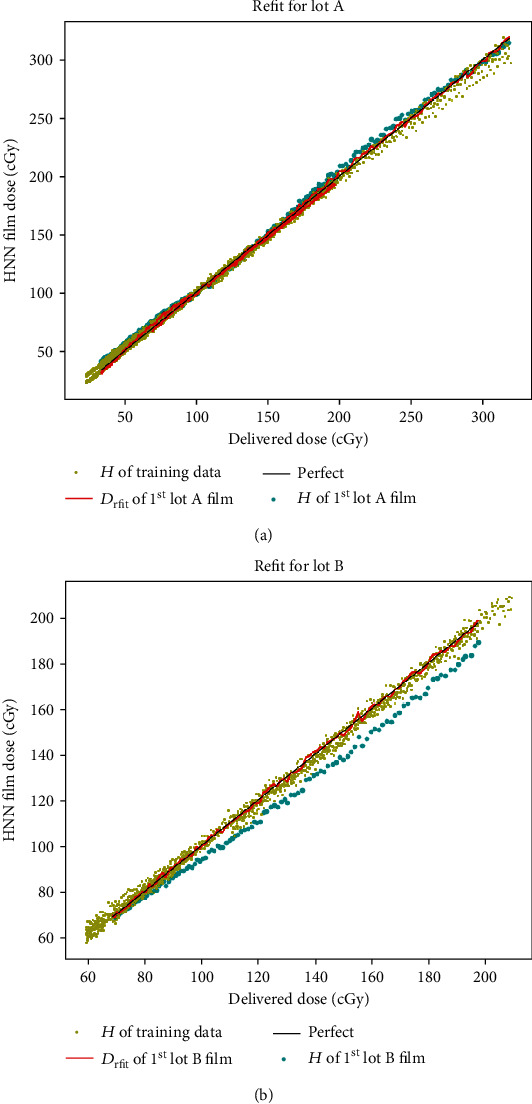
*H* values of training data (yellow dots), *D*_rfit_ by equation (5) (red line), “Perfect” black line (calculated doses equal to delivered doses), and the *H* values (blue dots) of 1^st^ calibration films of (a) lot A and (b) lot B.

Figure 5 illustrates the training results of the 2394 inputs from the portion I films of lot C, with the *H* values calculated through the trained HNN using the 1^st^ calibration film of lots A and B, and their refit doses calculated through equation (5). A black line, “Perfect”, shows the ultimate goal of calculated doses equal to delivered doses.

Since lots A and B are different from lot C, the *H* values (blue dots), calculated doses using the trained HNN, are apart from the black line (Figure 5). After the refitting procedure (equation (5)), the calculated dose (red line) approaches the black line.

**Figure 6:**
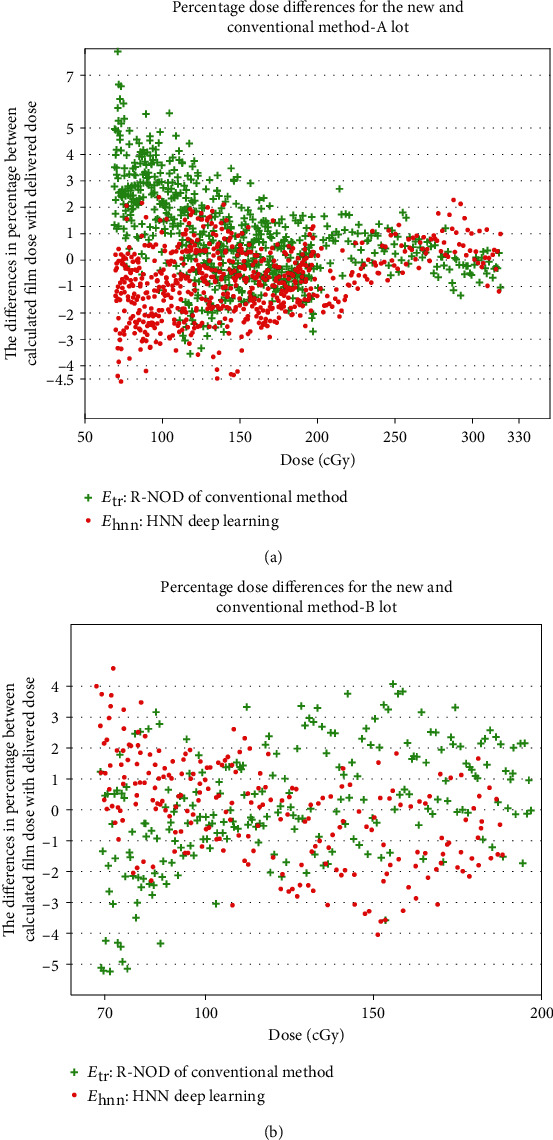
Percentage differences between the calculated dose (equation (2), equation (5)) and the delivered dose for the verification test of Lot A and Lot B films.

To calculate the fitting parameters of equations (2) and (5), 228 and 114 data points were extracted from the 1^st^ calibration film of lots A and B, respectively. For the verification test, the complete 684 and 228 inputs of lots A and B, respectively, excluding the 1^st^ calibration, were used to calculate *D*_rd_ (equation (6)) and compared with the delivered doses (Figure 6). The percentage error, *E*_hnn_, is within 4.5% using the deep learning HNN method. The percentage error, *E*_tr_, generally is also within 4.5%, by using the conventional R-NOD method, but would be higher than 7% and 5% for the delivered doses around 70 cGy, of lot A and lot B, respectively.

**Figure 7:**
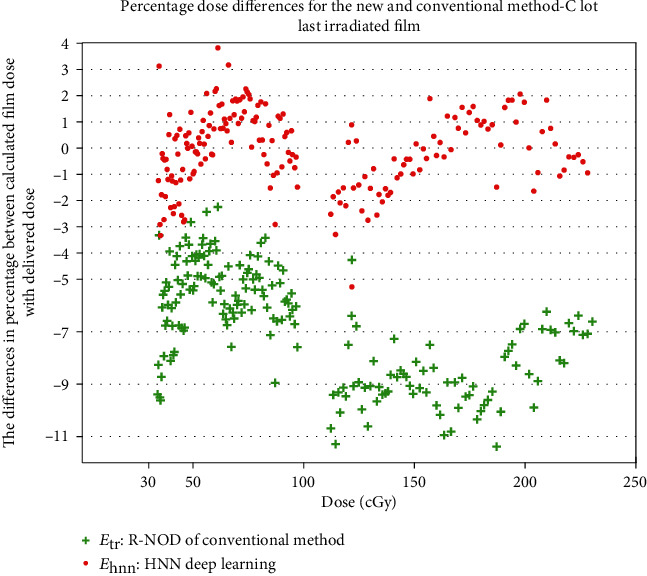
Percentage differences between the calculated dose and the delivered dose for the aging-effect verification test.

The 17^th^ calibration film of lot C is used for the verification test of the aging effect. The calculated dose through the trained HNN compared with the delivered dose is within 4% (Figure 7). The calculated dose through the R-NOD by equation (2), where the fitting parameters are obtained from the 16^th^ calibration film, four months before the 17^th^ calibration date, is generally lower than the delivered dose with a mean percentage error of 6.8%, though some exceed 11%. This higher percentage error is because of the aging effect, which means the recalibration is needed if using the conventional method. In contrast, the HNN method can help compensate for this effect.

The MSE calculated after the HNN training of portions I and II is 11.19 (2394 inputs) and 14.47 cGy (3762 inputs), MAE 2.39 cGy and 2.55 cGy, respectively. The averaged MSE of all the verification tests is less than 6.4 cGy. Compared with the study of Zhuang et al. using pytorch with 2-6-3-1 pretraining ANN and 2-6-3-9-1 protraining ANN, 400 NOD training inputs and 80 test NOD inputs [38], more training data and test data (thousands vs. hundreds), were used in our study. Our results also show a lower averaged verification test MSE, 6.4 cGy vs. 10.4 cGy.

Examining either high dose or low dose aspects, the two data ends shown in Figure 3 for the training process of our HNN model, it can be seen that if one part converges well, the other part will have divergences. The final chosen model is actually the compromise of the above. There are some trials that may improve the HNN modeling in future work: (1) modifying the activation functions, hidden layers, and the neuron numbers of the HNN model; (2) using R-NOD to separate the delivered doses to several ranges, with each range having its own well-trained HNN model; and (3) add new, appropriate parameters to the HNN model.

If the film used is not from the training lot, its *H* value generally will substantially depart from the “perfect” line (Figure 5) due to intralot variability of the film sensitivity, which requires physicists to calibrate new film lots at least once (by equation (5)). To apply our HNN model, equation (5) was used for the new lot refit, and it proved to be workable (Figure 6). However, equation (5) may not be feasible if the film sensitivity of a new lot varies so much that the *H* values will be far from the “perfect” line. Future research could consider resolving intralot variation, by putting the new lot calibration parameters into the HNN training model and giving them higher weights.

## 4. Conclusions

A deep leaning HNN method to calibrate the EBT3 film with better calibration accuracy than the conventional R-NOD method is presented. About the aging effect, the percentage error of the HNN method is within 4% and proved to be unaffected, while the averaged percentage error of the conventional R-NOD method is about 6.8%. This new technique can be improved by updating the new calibration data into the HNN training system whenever physicists perform the recalibration. Basing on collecting calibration data with the HNN method, physicists could spend less calibration time and reduce film usage.

## Data Availability

The data used to support the findings of this study are available from the corresponding author upon request.
